# Building bundles by the numbers

**DOI:** 10.7554/eLife.111840

**Published:** 2026-06-23

**Authors:** Christian Vanhille-Campos, Anđela Šarić

**Affiliations:** 1 Laboratoire Jean Perrin, CNRS / Sorbonne Université Paris France; 2 Institute of Science and Technology Austria Klosterneuburg Austria

**Keywords:** cytoskeleton, actin bundles, actin filaments, eukaryotic cells, cell protrusions, None

## Abstract

The size and shape of cytoskeletal bundles, essential regulators of cell function, emerge from collective filament assembly rather than precise size-control mechanisms.

**Related research article** Rosario A, McInally SG, Jelenkovic PR, Goode BL, Kondev J. 2026. Universal length fluctuations of actin structures found in cells. *eLife* **12**:RP91574. doi: 10.7554/eLife.91574.

Cells exhibit an outstanding diversity of shapes, reflecting a fundamental principle of multicellular life: specialisation. A single organism can contain hundreds of distinct cell types, each performing a unique biological role (Heintzman et al., 2009). In turn, each cell type’s specific shape is tailored to its function. For example, neurons extend elaborate branches to transmit information across long distances, while skin cells form flat protective layers.

Across biology, cellular architecture and function are intimately linked (Putra et al., 2023). A key determinant of this architecture is the cytoskeleton, the dynamic network of filaments often described as the cell’s “muscles and bones.” Among its components, actin filaments assemble into bundles of many nanometer-wide filaments, which are then cross-linked into larger structures. These bundles help cells change shape, often through forming membrane protrusions (Figure 1). The size and shape of these protrusions are adapted to specific tasks. As a result, actin filament bundles play a central role in cell architecture and function (Rajan et al., 2023).

**Figure 1.**
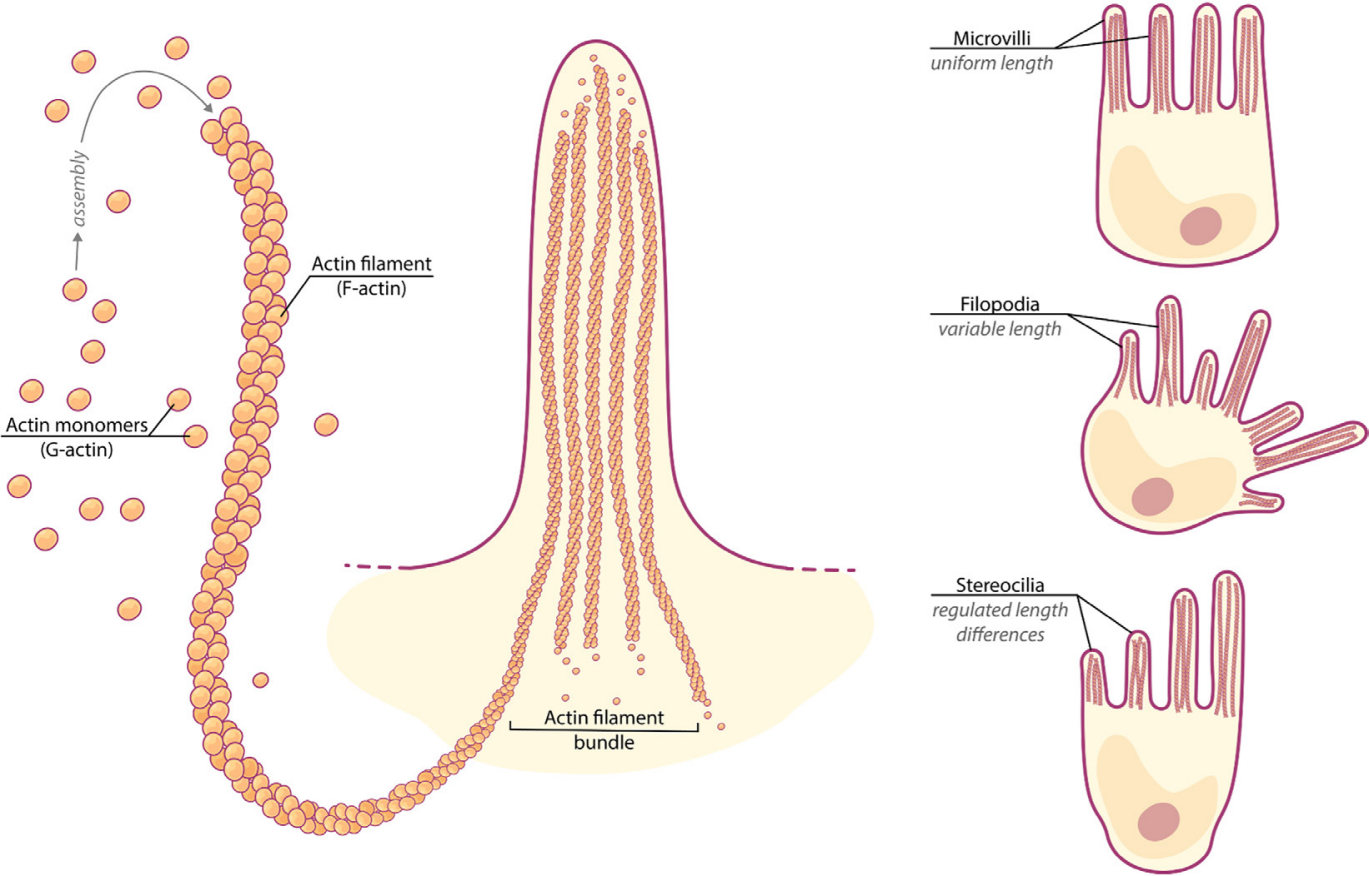
Actin-filament structures in cells. Left: Actin bundle schematic illustrating its multiscale and collective nature: actin monomers (orange bead) assemble into dynamic filaments that gather into a large membrane-deforming bundle. Right: Examples of cell shapes formed by actin bundles, whose precise size and shape are tightly linked to their function.

Image credit: Christian Vanhille-Campos and Claudia Flandoli (CC BY 4.0).

The importance of precise bundle architecture is evident across biology. In the inner ear, actin bundles form stereocilia, mechanosensitive protrusions that convert sound waves into electrical signals that our brains can process. These structures are arranged into staircases, whose width and height increments are essential for hearing, such that disruptions in their architecture can lead to hearing loss (Miyoshi et al., 2022). Filopodia, thin finger-like protrusions found at the leading edge of many motile cells, serve as sensors that probe the environment (Mattila and Lappalainen, 2008). Their length determines how far a cell can “reach” to detect cues, and defects in size control can impair migration, with important consequences for processes such as immune response (Zünd et al., 2025). Similarly, epithelial cells lining the small intestine are covered by microvilli, bundle-like projections that form a brush border. Their uniform height and specific arrangement are both critical for effective nutrient absorption and pathogen defence (Mosa et al., 2018). Across these diverse systems, filament bundles are not merely structural elements: their shape and particularly their size are critical regulators of function.

How actin bundles acquire their size and architecture has remained a long-standing question in science. Because these structures contain many interacting filaments – with molecular processes occurring across a broad range of spatial and temporal scales – linking bundle-scale properties to the underlying assembly dynamics has remained a major challenge. A common assumption has been that bundle size is controlled through length-dependent growth and shrinkage rates (so-called “balance-point” models), during which actin structures are constantly being rebuilt, with actin molecules and associated proteins rapidly added and removed (Chan and Marshall, 2012; Mohapatra et al., 2016).

Now, in eLife, Aldric Rosario, Shane McInally, Predrag Jelenkovic, Bruce Goode and Jane Kondev report new insights on filament length regulation (Rosario et al., 2026). The researchers, based at Brandeis University and Columbia University, propose that each filament grows independently and that the bundle’s length is determined by its longest filament. To test this theory, they used computer models to simulate polymer and bundle lengths and shape, reexamining experimental measurements of the lengths of stereocilia, microvilli, actin cables and filopodia .

Rosario et al. show that the length-dependent growth and shrinkage rates mechanism is inconsistent with previously published experiments across a wide range of bundle types and cell systems. Their statistical analysis of available data across biological systems revealed that filament lengths cannot be explained by balance-point models, pointing instead to a different underlying mechanism.

Rosario et al. demonstrate the importance of collective effects in controlling bundle size, which depend on the statistical properties of the filament population rather than on the characteristics of any single individual. By treating bundles as assemblies of many interacting filaments, they developed a theoretical framework that accurately predicts key architectural properties, including bundle lengths and width profiles. Remarkably, the predicted statistical signatures of bundle length distributions quantitatively match previous experimental observations. The results revealed that cells do not need explicit mechanisms to control bundle size; instead, actin bundle architecture can emerge naturally from the collective dynamics of many filaments.

More broadly, they established a clear statistical link between the properties of individual filaments and the large-scale characteristics of the bundles they form. This connection offers a powerful new route for probing cytoskeletal systems without relying on experimentally demanding measurements at high spatial and temporal resolution. Looking ahead, it will be exciting to extend this approach beyond static structural features to dynamic properties of filament bundles and other multibody cytoskeletal assemblies. Can different underlying filament dynamics be distinguished through their collective signatures? How do the lifetimes of these structures emerge from the dynamics of their constituent filaments? Addressing such questions will no doubt provide valuable new insight into how cells build, maintain, and regulate the complex architectures that underpin life.
